# A novel plant growth-promoting rhizobacterium, *Rhizosphaericola mali* gen. nov., sp. nov., isolated from healthy apple tree soil

**DOI:** 10.1038/s41598-024-51492-y

**Published:** 2024-01-10

**Authors:** Han Sol Kim, Ji-Sun Kim, Min Kuk Suh, Mi Kyung Eom, Jiyoung Lee, Jung-Sook Lee

**Affiliations:** 1 Korean Collection for Type Cultures, Korea Research Institute of Bioscience and Biotechnology, 181 Ipsin-gil, Jeongeup-si, Jeollabuk-do 56212 Republic of Korea; 2 Department of Lifestyle Medicine, Jeonbuk National University, 79 Gobong-ro, Iksan-si, Jeollabuk-do 54596 Republic of Korea; 3 University of Science and Technology (UST), 217 Gajeong-ro, Yuseong-gu, Daejeon, 34113 Republic of Korea

**Keywords:** Ecology, Microbiology

## Abstract

The rhizosphere microbial community is closely associated with plant disease by regulating plant growth, agricultural production, nutrient availability, plant hormone and adaptation to environmental changes. Therefore, it is very important to identify the rhizosphere microbes around plant roots and understand their functions. While studying the differences between the rhizosphere microbiota of healthy and diseased apple trees to find the cause of apple tree disease, we isolated a novel strain, designated as B3-10^T^, from the rhizosphere soil of a healthy apple tree. The genome relatedness indices between strain B3-10^T^ and other type species of family *Chitinophagaceae* were in the ranges of 62.4–67.0% for ANI, 18.6–32.1% for dDDH, and 39.0–56.6% for AAI, which were significantly below the cut‑off values for the species delineation, indicating that strain B3-10^T^ could be considered to represent a novel genus in family *Chitinophagaceae.* Interestingly, the complete genome of strain B3-10^T^ contained a number of genes encoding ACC-deaminase, siderophore production, and acetoin production contributing to plant-beneficial functions. Furthermore, strain B3-10^T^ was found to significantly promote the growth of shoots and roots of the *Nicotiana benthamiana*, which is widely used as a good model for plant biology, demonstrating that strain B3-10^T^, a rhizosphere microbe of healthy apple trees, has the potential to promote growth and reduce disease. The phenotypic, chemotaxonomic, phylogenetic, genomic, and physiological properties of this plant growth-promoting (rhizo)bacterium, strain B3-10^T^ supported the proposal of a novel genus in the family *Chitinophagaceae*, for which the name *Rhizosphaericola mali* gen. nov., sp. nov. (= KCTC 72123^T^ = NBRC 114178^T^).

## Introduction

The rhizosphere refer to the boundary between plant roots and soil, which is a narrow soil area that interacts with and surrounds the plant roots^1^. Rhizosphere is an environment that affects the growth and health of plants, and involves a wide variety of microorganisms^2^. The various microorganisms, including bacteria, fungi, and mycorrhizae, are collectively known as the rhizosphere microbiome and are referred to as the important second genomes of plants^3^. In addition, the rhizosphere microbiome can enhance plant immune functions^4^. Therefore, understanding how the rhizosphere microbiome affects plant performance is of great agronomic importance.

The rhizosphere microbiome can be classified as microorganisms that are deleterious, beneficial or neutral to plants^5^. Microorganisms that have been reported to have detrimental effects on the plant growth and health include pathogenic fungi, oomycetes, pathogenic bacteria, and nematodes, while beneficial microorganisms include nitrogen-fixing bacteria, endo- and ectomycorrhizal fungi, and plant growth-promoting bacteria (PGPB)^6^. Harmful microbes, such as soilborne pathogens, cause yield loss and threaten agricultural production^7^. However, beneficial microoganisms, including mutualistic microbes, can enhance plant growth by increasing nutrient availability, producing plant hormones to increase tolerance to abiotic stresses, and adapting to environmental changes^8–12^. The plant growth-promoting (rhizo)bacteria (PGPB or PGPR) produce compounds that enhance plant growth, increase root surface area for nutrient uptake in the soil, and play an important role in macro- and micro-nutrient cycling^13^. Substances produced by PGPB, such as indole acetic acid, cytokinins, and siderophores, are responsible for regulating plant growth and phytohormones, and protecting plants from harmful microorganisms^14,15^.

The family *Chitinophagaceae* of the class *Chitinophagia*, belonging to the phylum *Bacteroidetes*, was proposed by Kämpfer et al. and currently consists of 50 genera with valid published names^16,17^. Members of the family *Chitinophagaceae* are Gram-stain-negative, aerobic or facultatively anaerobic, and have been isolated mostly from rhizospheric soils, sediments, marines, and water of lakes. In addition, the major respiratory quinone is MK-7 and the major polar lipid is phosphatidylethanolamine. The major cellular fatty acids are iso-C_15:0,_ iso-C_15:1_ G, and iso-C_17:0_ 3OH. It has been reported that many species of family *Chitinophagaceae* had the ability to fix nitrogen in the rhizosphere of plants and inhibit the growth of plant pathogens^18,19^. Some strains have been reported to have plant growth-promoting (PGP) properties by possessing 1-aminocyclopropane-1-carboxylate (ACC) deaminase and PGP-related genes^20,21^, indicating the possibility that strains belonging to the family *Chitinophagaceae* can express PGP-related genes to promote plant growth.

In this study, we report on the taxonomic, physiological, genomic, and PGP characteristics of a novel strain B3-10^T^ isolated from rhizosphere soil. On the basis of the phenotypic, chemotaxonomic, and phylogenetic data, strain B3-10^T^ represents a novel genus in the family *Chitinophagaceae*, with the proposed name, *Rhizosphaericola mali* gen. nov., sp. nov.

## Results and discussion

### 16S rRNA gene sequencing and phylogenetic analyses

**Figure 1:**
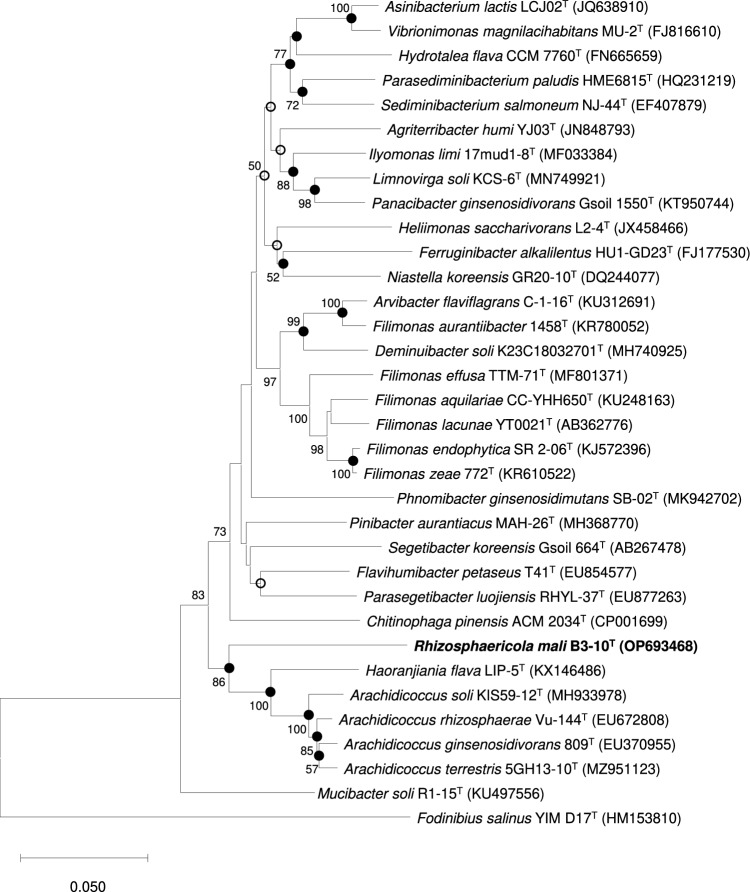
Neighbor-joining (NJ) tree based on 16S rRNA gene sequences showing the relationship between strain B3-10^T^ and closely related species of family *Chitinophagaceae*. Bootstrap values > 50% are given at the nodes. Filled circles indicate nodes that were also recovered in the maximum-likelihood and maximum-parsimony algorithms. Open circles indicate nodes that were also found with the maximum-likelihood algorithm. *Fodinibius salinus* YIM D17^T^ was used as an outgroup. Bar, 0.05 substitutions per nucleotide position.

The 16S rRNA gene sequence analysis revealed that strain B3-10^T^ had the highest similarity of 89.5% to *Arachidicoccus soli* KIS59-12^T^ within the family *Chitinophagaceae*, which was much lower than the 98.7% that generally defines bacterial species^22^. Phylogenetic tree based on 16S rRNA gene sequences also showed that strain B3-10^T^ belonged to the family *Chitinophagaceae* and formed a separate clade away from other members of the family *Chitinophagaceae* (Fig. 1, Supplementary Fig. S1). In addition, the 16S rRNA similarity values between strain B3-10^T^ and strains forming clusters with strain B3-10^T^ in phylogenetic tree were 89.4% with *A. rhizosphaerae* Vu-144^T^, 89.3% with *A. terrestris* 5GH13-10^T^, 89.0% with *A. ginsenosidivorans* Gsoil 809^T^, and 87.8% with *Haoranjiania flava* LIP-5^T^. The results for 16S rRNA gene sequencing and phylogenetic analyses indicated that strain B3-10^T^ belonged to novel genus within the family *Chitinophagaceae*.

### Whole genome sequencing and phylogenomic analysis

The complete genome of strain B3-10^T^ had a total length of 4,701,441 bp, which consisted of one chromosome of 4,671,108 bp and one plasmid of 30,333 bp. The sequencing depth of coverage was 423.0x. Its genome contained 4072 predicted genes, including 3,953 protein-coding gene and 51 pseudogenes. It also contained 53 tRNAs and 12 rRNAs (Four 5S, 16S, and 23S rRNA genes each) genes. The DNA G + C content of strain B3-10^T^ was 34.2 mol%, which was the lowest value among the type strains of the family *Chitinophagaceae* (38.2–51.6 mol%). The genomic features and graphical circular genome map of strain B3-10^T^ are shown in Supplementary Table S1 and Supplementary Fig. S2, respectively.

**Figure 2:**
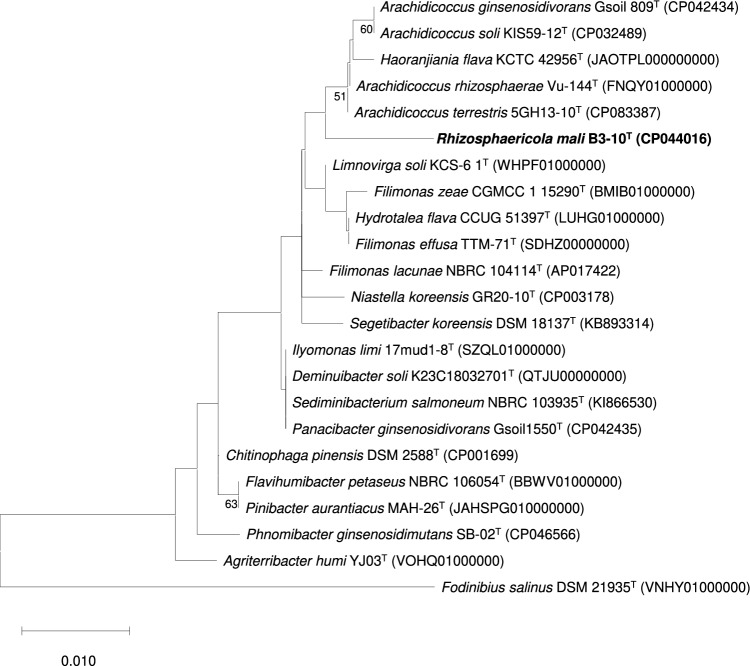
Maximum-likelihood (ML) tree based on whole genome sequences showing the relationship between strain B3-10^T^ and closely related species of family *Chitinophagaceae*. Bootstrap values over > 50% are shown on the nodes as percentages of 1000 replicates. *Fodinibius salinus* YIM D17^T^ was used as an outgroup. Bar, 0.01 substitutions per nucleotide position.

The genome relatedness indices between strain B3-10^T^ and other type species of family *Chitinophagaceae* were in the ranges of 62.4–67.0% for ANI and 18.6–32.1% for dDDH (Supplementary Table S2), which was below the species threshold (95–96% and 70%, respectively)^23^. In addition, AAI value is considered as a more reliable parameter for discriminating higher ranks than species^24^. The calculated AAI values between strain B3-10^T^ and other type species of family *Chitinophagaceae* were ranged from 39.0 to 56.6% (Supplementary Table S2), which was below 65%, the threshold proposed for different genus^25^. These values revealed that strain B3-10^T^ represented a lineage at a higher taxonomic rank. Furthermore, the whole-genome phylogenetic tree constructed using the ML method showed that strain B3-10^T^ was clearly separated from other genera of the family *Chitinophagaceae* (Fig. 2). These results demonstrated that strain B3-10^T^ represents a novel species in a novel genus of the family *Chitinophagaceae.*

**Figure 3:**
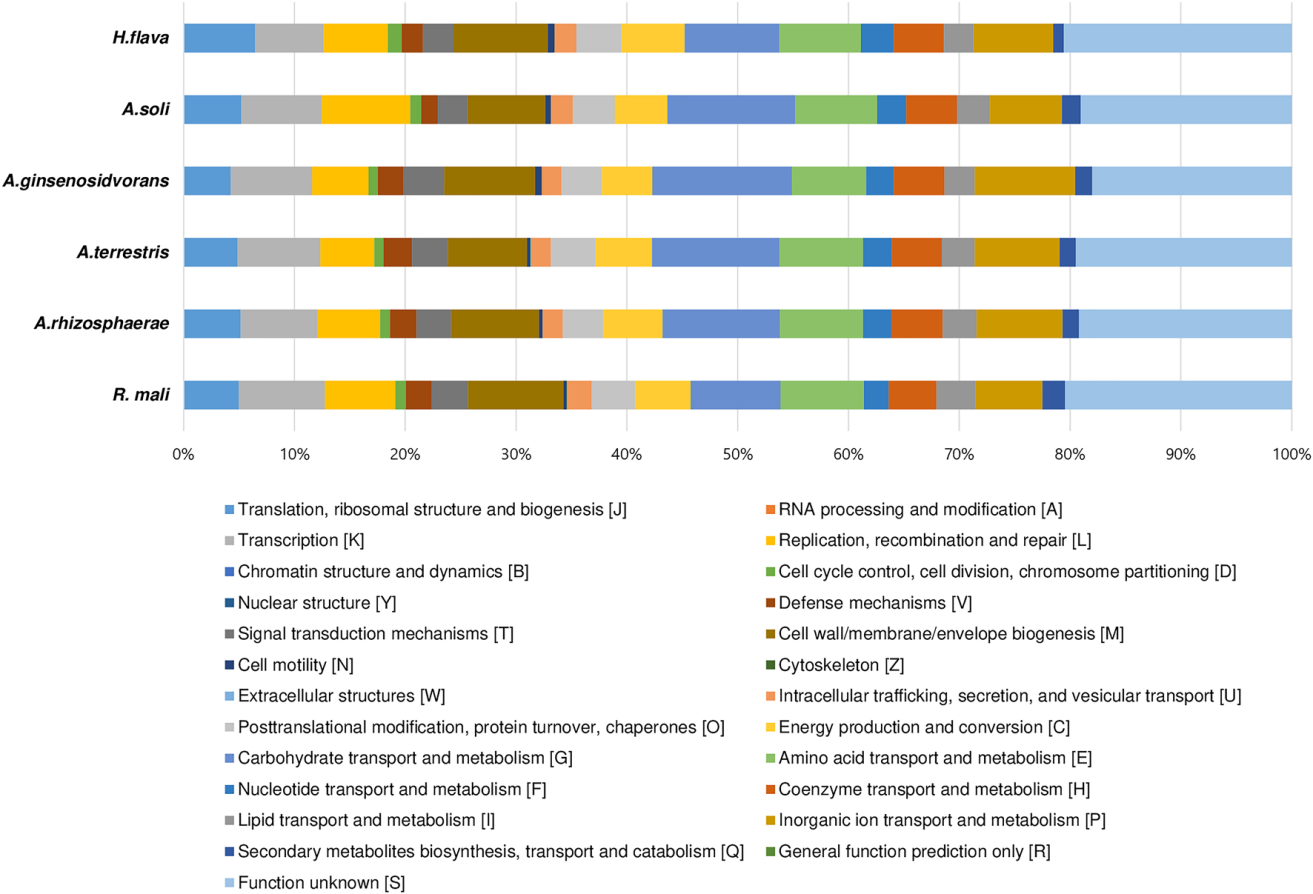
Gene distribution based on 25 general eggNOG functional categories predicted in the genome of strain B3-10^T^.

The analysis of genome dataset using the eggNOG (evolutionary gene genealogy Non-supervised Orthologous Groups) database revealed the presence of a total of 3,725 clusters of orthologous genes (COGs), which were further categorized into 24 distinct functional categories (Fig. 3). Among the obtained functional groups, the cluster for S (function unknown: 1339 genes) constituted the largest functional group. Among the other functional groups, the clusters for M (cell wall/membrane/envelope biogenesis; 248 genes), K (Transcription; 225 genes), G (carbohydrate transport and metabolism; 218 genes), and E (amino acid transport and metabolism; 204 genes) were the most highly represented categories (in descending order).

### Morphological, physiological, and biochemical analyses

**Figure 4:**
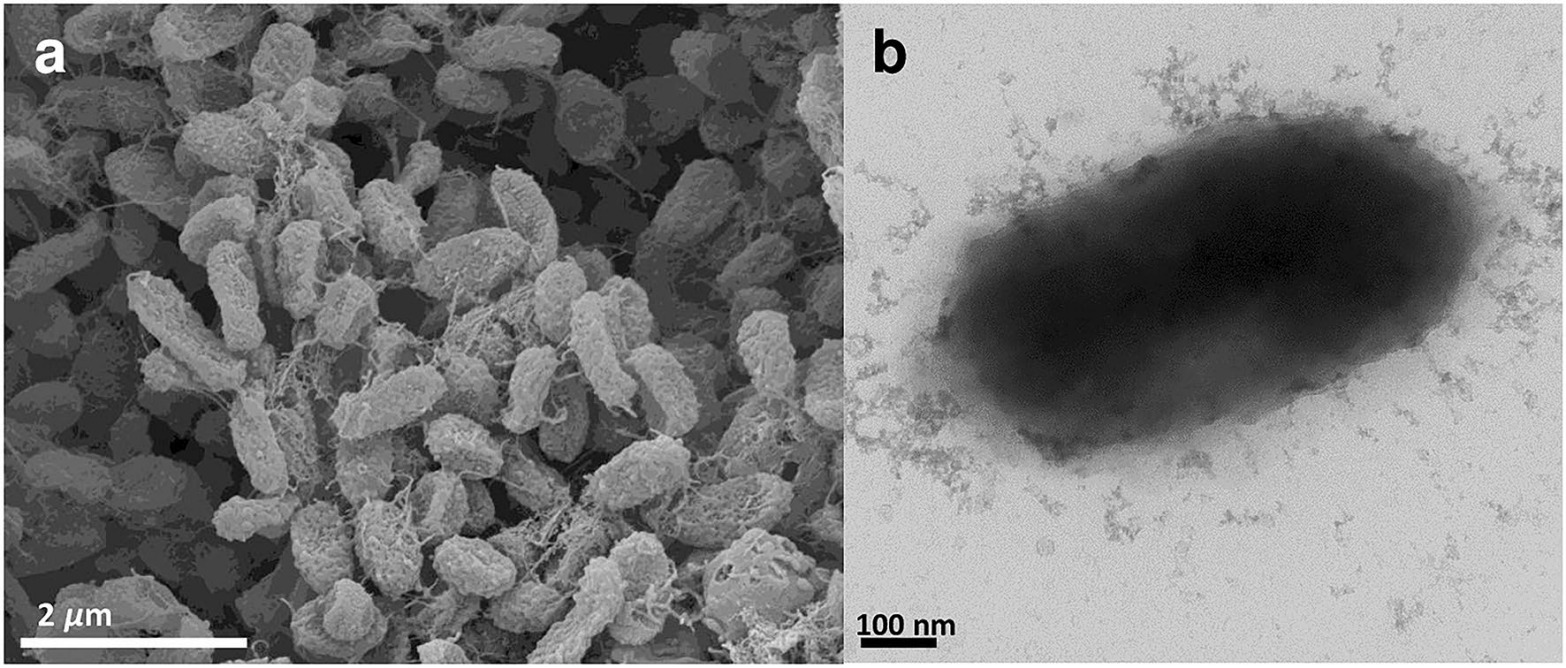
Scanning electron micrograph (**a**) and transmission electron microscopy (**b**) of strain B3-10^T^ grown on R2A at 30 °C for 3 days.

Cells of strain B3-10^T^ were Gram-stain-negative, strictly aerobic, non-motile, and short rod with 0.6–1.5 µm long and 0.5–0.6 µm wide (Fig. 4). Colonies of strain B3-10^T^ on R2A agar plates after 3 days of incubation at 30 °C were circular with white, convex, and moist smooth. Growth occurred at temperatures ranging from 10 to 30 °C (optimum, 30 °C), at pH 5.0–8.0 (optimum, pH 7.0), and in the presence of 0–2.0% NaCl (w/v; optimum, 0%). Positive for catalase and negative for oxidase. In the API 20NE, positive for *β*-glucosidase (esculin hydrolysis), *β*-galactosidase (PNPG), d-glucose, l-arabinose, d-mannose, *N*-acetyl-d-glucosamine, and d-maltose; negative for Indole production, glucose acidification, arginine dihydrolase, urease, protease (gelatin hydrolysis), d-mannitol, gluconate, caprate, adipate, malate, citrate, and phenyl-acetate. In the API 20E, positive for 2-nitrophenyl-*β*-d-galactopyranoside, sodium pyruvate, d-glucose, d-melibiose, amygdalin, and l-arabinose; weakly positive for gelatin, l-rhamnose, d-sucrose, and amygdalin; negative for l-arginine, l-lysine, l-ornithine, trisodium citrate, sodium thiosulfate, urea, l-tryptophane, l-tryptophane, d-mannitol, inositol, and d-sorbitol. According to API ZYM test results, positive for alkaline phosphatase, esterase (C4), leucine arylamidase, valine arylamidase, acid phosphatase, naphtol-AS-BI-phosphohydrolasem, *α*-galactosidase, *β*-galactosidase, *α*-glucosidase, *β*-glucosidase, *N*-acetyl-*β*-glucosaminidase, and *α*-fucosidase; weakly positive for esterase lipase (C8); negative for lipase (C14), cystine arylamidase, trypsin, *α*-chymotrypsin, *β*-glucuronidase, and *α*-mannosidase. The distinctive phenotypic characteristics of strain B3-10^T^ and the reference strains were listed in Supplementary Table S3. Above results showed that distinctive physiological and biochemical characteristics of strain B3-10^T^ with closely related genera in the family *Chitinophagaceae.* Strain B3-10^T^ showed distinct differences from the genus *Haoranjiania* in α-mannosidase and *N*-acetyl-d-glucosamine activities and from members of the genus *Arachidicoccus* in α-glucosidase activity. Furthermore, morphological and physiological comparisons between strain B3-10^T^ and other type species in the family *Chitinophagaceae* are in Supplementary Table S4.

### Chemotaxonomic characteristics

The major fatty acids from strain B3-10^T^ were summed feature 3 (C_16:1_ *ω*6c and/or C_16:1_ *ω*7c; 28.0%), iso-C_15:0_ (25.7%), and iso-C_15:1_ G (25.5%). For comparison, the fatty acids of *A. rhizosphaerae* KCTC 22378^T^ were iso-C_15:0_ (45.9%), iso-C_17:0_3OH (16.5%), summed feature 3 (C_16:1_ *ω*6c and/or C_16:1_ *ω*7c; 11.0%), and iso-C_15:1_G (10.3%); *A. terrestris* KCTC 92783^T^ were iso-C_15:0_ (26.6%), iso-C_15:1_G (21.8%), summed feature 3 (C_16:1_ *ω*6c and/or C_16:1_ *ω*7c; 18.1%), and iso-C_17:0_3OH (16.8%); *A*. *ginsenosidivorans* KCTC 22820^T^ were iso-C_15:0_ (42.4%), iso-C_15:1_G (14.5%), iso-C_17:0_3OH (14.2%), and C_16:0_ (10.0%); *A. soli* KCTC 92782^T^ were iso-C_15:0_ (38.9%), iso-C_17:0_3OH (18.4%), and iso-C_15:1_G (18.2%); *H*. *flava* KCTC 42956^T^ were iso-C_15:0_ (33.5%), and anteiso-C_15:0_ (27.3%) were revealed as major fatty acids (Supplementary Table S5). The fatty acid analysis revealed distinctive characteristics of strain B3-10^T^ from closely related type species in the family *Chitinophagaceae.* Summed Feature3, one of the major fatty acids, was more abundant in strain B3-10^T^ (28.0%) compared to members of the genus *Arachidicoccus* (9.1–18.1%) and the genus *Haoranjiiania* (7.7%). The fatty acid iso-C_17:0_ 3OH was minor in strain B3-10^T^ (4.8%), while it was major in members of the genus *Arachidicoccus* (14.2–18.4%) and absent in the genus *Haoranjiiania*. Additionally, anteiso-C_15:0_ was minor fatty acid in strain B3-10^T^ (1.6%) and the genus *Arachidicoccus* (1.1–2.5%), but was a major fatty acid in the genus *Haoranjiiania* (27.3%).

The respiratory quinine of strain B3-10^T^ was MK-7. The polar lipids of strain B3-10^T^ were phosphatidylethanolamine (PE), two unidentified lipids (Ls), an unidentified aminolipids (ALs), three unidentified phospholipid (PL), and three unidentified aminophospholipids (APLs) (Supplementary Fig. S3).

### Plant growth-promoting characteristics

As demonstrated by many research findings, the pivotal role of plant growth-promoting bacteria (PGPB) in facilitating plant growth is largely due to their ACC-deaminase activity^26^. Excessive levels of ethylene, a known inhibitor of plant growth^27^, can be counteracted by the hydrolytic activity of ACC-deaminase toward ACC, a precursor of ethylene, resulting in reduced plant ethylene concentrations^28^. Furthermore, ACC-deaminase can effectively protect plants from various environmental stresses, including drought and salinity stresses^29^. This vital enzymatic activity is reported to be mediated by the *dycD* and *rimM* genes encoding ACC-deaminase^30^. It was confirmed that *dcyD* and *rimM* genes were present in strain B3-10^T^ genome, showed that strain B3-10^T^ was able to produce ACC-deaminase. Siderophore production of PGPB is known to enhance the uptake and utilization of iron to promote plant growth^31^. Iron is essential for plant growth as it is required for the process of N_2_ fixation in plants^32^. Iron usually exists in soil as divalent (Fe^2+^) and trivalent (Fe^3+^) cations, and plants uptake their ions depends on several factors, including soil pH and levels of other soil nutrients^33^. Siderophores can chelate heavy metals such as Al, Cd, Cu, Pb, and Zn to reduce metal toxicity to plants^34^. Furthermore, in the genome of strain B3-10^T^, seven *fhuA* homologues, *fbpC, feoB*, and *exbB* were identified. The *fhuA* encodes a TonB-dependent siderophore receptor and is involved in ferric hydroxamate uptake and the transport of ferrichrome^35^. The *fecI* is a ferric citrate transporter, *fbpC* is involved in Fe^3+^ uptake, and *feoB* is involved in Fe^2+^ uptake, and *exbB* transports iron-containing siderophores from the bacterial outer membrane into the periplasmic space^36–38^. Furthermore, the genome of strain B3-10^T^ encoded *alsS*, *alsD*, and *butA* related to acetoin production. Volatile substances such as 2,3-butanediol and 3-hydroxy-2-butanone (acetoin) produced by PGPB have been reported to promote plant growth^39^. Acetoin is synthesized by the enzymes acetolactate synthase (AlsS) and acetolactate decarboxylase (AlsD), which are encoded by the genes *alsS* and *alsD*. To synthesize 2,3-butanediol, pyruvate is converted to acetolactate by AlsS, and acetolactate is decarboxylated to acetoin by AlsD^40^. Acetolactate can be spontaneously converted to diacetyl (2,3-butanedione), which in turn can be converted to acetoin by diacetyl reductase (*butA*)^41^. These genomic analyses showed that strain B3-10^T^ possesses many genes related with PGP ability (Supplementary Table S6). Therefore, we determined whether strain B3-10^T^ possessed plant growth-promoting abilities contributed by these PGP-related genes.

**Figure 5:**
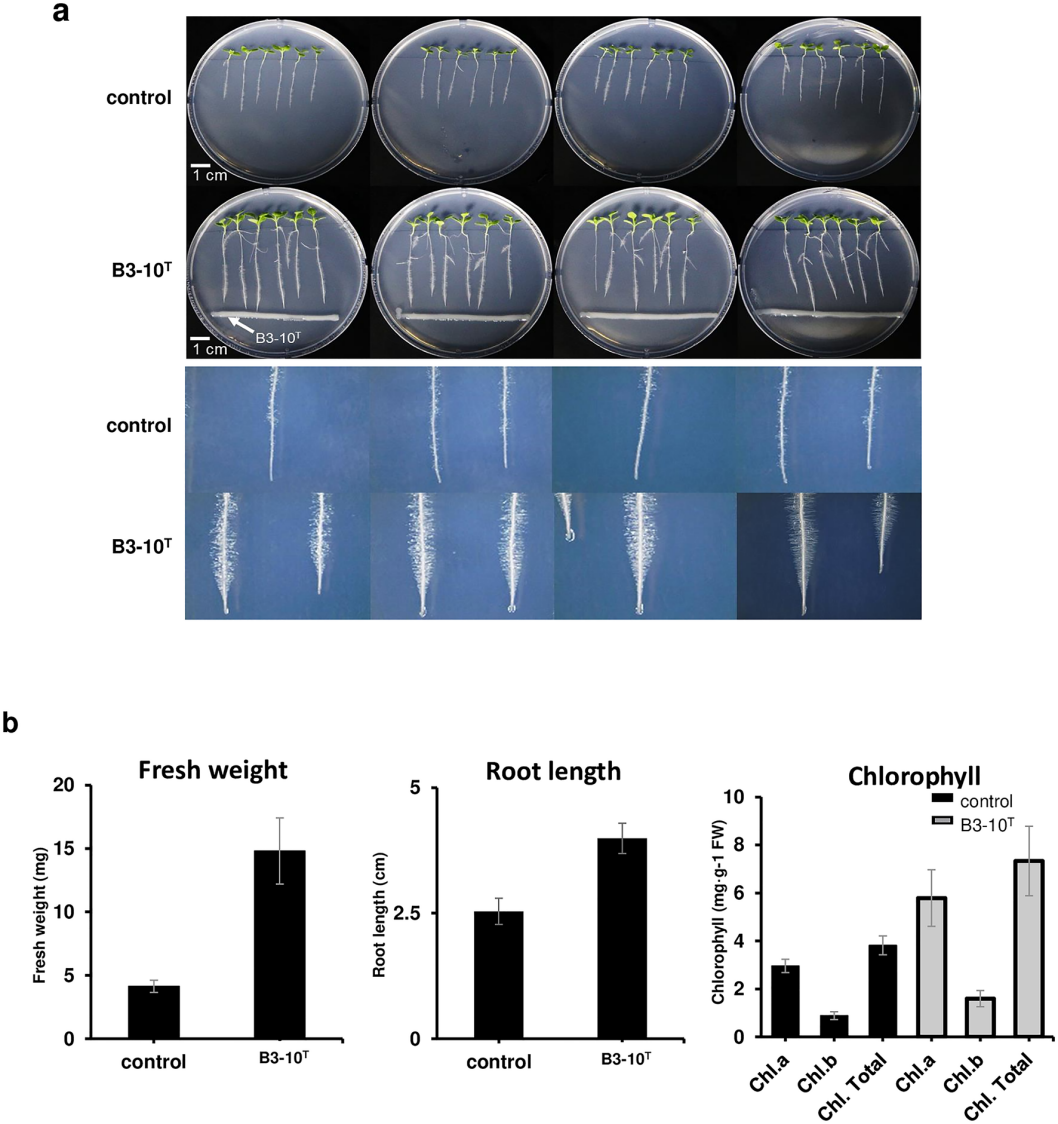
Effect of strain B3-10^T^ on growth and development of *N. benthamiana.* (**a**) *N. benthamiana* were grown for 14 days on 1/2 MS agar plates. Plant samples inoculated with strain B3-10^T^ on agar plates and non-inoculated plant samples (control) were compared. (**b**) The fresh weight (mg), root length (cm), and chlorophyll content (mg g^−1^ FW) of *N. benthamiana* plants were measured after 14 days of inoculation with strain B3-10^T^. Data show mean ± standard deviation of 6 plants.

The activity of ACC-deaminase was determined by measuring α-ketobutyrate production in the presence of ACC. Strain B3-10^T^ produced more α-ketobutyrate by ACC supplementation, indicating that strain B3-10^T^ possessed ACC-deaminase activity to degrade ACC and produce α-ketobutyrate (Supplementary Table S7). Moreover, the orange halo on chrome azurol S (CAS) agar showed that strain B3-10^T^ produced siderophore (Supplementary Fig. S4). Based on genomic analysis and activity test, it was examined whether strain B3-10^T^ was able to promote growth of *N. benthamiana,* which is widely used as a good model for plant biology. As the results, when strain B3-10^T^ was inoculated in plates with *N. benthamiana*, it was observed to increase the growth of the shoot and root of the *N. benthamiana* plants compared to the controls (Fig. 5a). Furthermore, the presence of strain B3-10^T^ increased the fresh weight of the plants by 3.6-fold compared to the controls, while the root length and the chlorophyll contents increased by 1.6-fold and 1.9-fold, respectively (Fig. 5b).

These results demonstrated that strain B3-10^T^ significantly promoted the growth of *N. benthamiana*, likely due to the presence of PGP-related genes. These results suggest that the presence of strain B3-10^T^ has a positive effect on plant growth and development.

## Conclusions

Strain B3-10^T^ was isolated from rhizosphere soil of healthy apple tree in Chungju-si, Chungcheongbuk-do, Republic of Korea. The phylogenetic and phylogenomic analysis showed that strain B3-10^T^ occupied a distinct position in the family *Chitinophagaceae* of the class *Chitinophagia*, clearly separated from other genera. The genome relatedness indices between strain B3-10^T^ and other type species of the family *Chitinophagaceae* are lower than the values that generally defines bacterial species. Strain B3-10^T^ was a Gram-stain-negative, non-spore-forming, non-motile, rod-shaped bacterium, similar to members of the family *Chitinophagaceae*, whose major respiratory quinone is MK-7 and whose major polar lipid is phosphatidylethanolamine. However, strain B3-10^T^ was distinguished from members of closely related genera in the family *Chitinophagaceae* with respect to phenotypic and biochemical properties, including carbohydrate metabolism, fatty acids composition, and DNA G + C content. The functional genome analysis and PGP ability test strongly suggest that strain B3-10^T^ can have a positive effect on plant growth and development by expressing many PGP-related genes.

Overall, on the basis of distinct phenotypic, phylogenetic, genomic features, and chemotaxonomic properties strain B3-10^T^ is represents a novel species of a new genus, for which the name *Rhizosphaericola mali* gen. nov., sp. nov. is proposed.

### Description of *Rhizosphaericola* gen. nov.

*Rhizosphaericola* (Rhi.zo.sphae.ri'co.la. Gr. fem. n. *rhiza*, root; Gr. fem. n. *sphaira*, sphere; L. suff. -*cola*, inhabitant, dweller; N.L. masc. n. *Rhizosphaericola*, an inhabitant of the rhizosphere).

Gram-stain-negative, non-spore-forming, non-motile, rod-shaped, and strictly aerobic bacterium. The range in which growth occurs is 10–30 °C (optimum, 30 °C) for temperature, 5.0–8.0 (optimum, pH 7.0) for pH, and 0–2.0% (w/v; optimum, 0%) for NaCl. The predominant fatty acids consisted of iso-C_15:0_, iso-C_15:1_ G, and summed feature 3 (C_16:1_ *ω*6c and/or C_16:1_ *ω*7c). The major respiratory menaquinone and phospholipid are MK-7 and phosphatidylethanolamine (PE), respectively. Belongs to the family *Chitinophagaceae*, order *Chitinophagales*, class *Chitinophagia*.

### Description of *Rhizosphaericola mali* sp. nov.

*Rhizosphaericola mali* (ma’li. L. gen. n. *mali*, of an apple, referring to the apple tree).

Gram-stain-negative, strictly aerobic, non-spore-forming, non-motile, and rod-shaped bacterium, 0.64–1.49 µm in length and 0.46–0.59 µm in diameter. Growth occurred at temperatures ranging from 10 to 30 °C (optimum, 30 °C), at pH 5.0–8.0 (optimum, pH 7.0), and in the presence of 0–2.0% NaCl (w/v; optimum, 0%). Positive for catalase and negative for oxidase. In the API 20NE, positive for *β*-glucosidase (esculin hydrolysis), *β*-galactosidase (PNPG), d-glucose, l-arabinose, and d-mannose; negative for Indole production, glucose acidification, arginine dihydrolase, urease, protease (gelatin hydrolysis), d-mannitol, *N*-acetyl-d-glucosamine, d-maltose, gluconate, caprate, adipate, malate, citrate, and phenyl-acetate. In the API 20E, positive for 2-nitrophenyl-*β*-d-galactopyranoside, sodium pyruvate, d-glucose, d-melibiose, amygdalin, and l-arabinose; weakly positive for gelatin, l-rhamnose, d-sucrose, and amygdalin; negative for l-arginine, l-lysine, l-ornithine, trisodium citrate, sodium thiosulfate, urea, l-tryptophane, l-tryptophane, d-mannitol, inositol, and d-sorbitol. According to API ZYM test results, positive for alkaline phosphatase, esterase (C4), leucine arylamidase, valine arylamidase, acid phosphatase, naphtol-AS-BI-phosphohydrolasem, *α*-galactosidase, *β*-galactosidase, *α*-glucosidase, *β*-glucosidase, *N*-acetyl-*β*-glucosaminidase, and *α*-fucosidase; weakly positive for esterase lipase (C8); negative for lipase (C14), cystine arylamidase, trypsin, *α*-chymotrypsin, *β*-glucuronidase, and *α*-mannosidase. The identified membrane phospholipids include phosphatidylethanolamine(PE). The unidentified lipids include 1 amino-, 3 phospho-, and 3 aminophospho -lipids. The fatty acid included (in order of abundance) iso-C_15:0_, iso-C_15:1_ G, and summed feature 3 (C_16:1_ *ω*6c and/or C_16:1_ *ω*7c). The respiratory menaquinons included MK-7. The G + C content of the DNA is 34.2% (genome). The type strain (B3-10 ^T^ = KCTC 72123^T^ = NBRC 114178^T^) was isolated from soil sample collected from apple orchard in Chungju-si, Chungcheongbuk-do, Republic of Korea. The 16S rRNA gene sequence and the genome sequence accession numbers in the GenBank are OP693468 and CP044016 (chromosome; CP044017, plasmid), respectively.

## Materials and methods

### Sampling site and isolation procedure of bacteria

The strain B3-10^T^ was isolated from rhizosphere soil samples around apple trees. The apple orchard from which soil samples were collected is located in Chungju-si, Chungcheongbuk-do, Republic of Korea (36°58′20.9″ N 127°57′31.7″ E). Samples were serially diluted with sterile distilled water, spread on Reasoner’s 2 Agar (R2A; BD) and incubated at 15 °C for 4 weeks. Single colonies were isolated and sub-cultured three times on fresh R2A plates. The single isolates were preserved in glycerol (10%, v/v) at − 80 °C for storage.

### 16S rRNA gene sequencing and phylogenetic analysis

The 16S rRNA gene sequences were analyzed to determine the phylogenetic position of strain B3-10^T^. For 16S rRNA gene sequencing, extraction of genomic DNA was performed using a commercial genomic DNA extraction kit (Biofact) according to the manufacturer's instructions. The 16S rRNA gene was amplified with the following universal bacterial primers: 27F (5′-AGAGTTTGATCCTGGCTCAG-3′), 518F (5′-CCAGCAGCCGCGGTAATAC-3′), 805R (5′-GACTACCAGGGTATCTAATC-3′), and 1492R (5′-TACGGYTACCTTGTTACGACTT-3′)^42^. The PCR product purified using PCR purification kit (Biofact) was sequenced directly from Biofact (Daejeon, Republic of Korea) using ABI 3730XL capillary DNA analyzer (Applied Biosystems) by fluorescent dye terminator method. The full length sequences of the 16S rRNA gene were aligned using the BioEdit program^43^. In addition, comparison of the 16S rRNA gene sequences (1454 bp and 1521 bp, respectively) obtained from Sanger sequencing and whole genome sequences showed that they were more than 99.9% identical. Therefore, we performed further studies on sequence similarity analysis and phylogenetic tree construction using 16S rRNA gene sequences from whole genome sequences.

The sequence similarity analysis was compared with sequences retrieved from the EzBioCloud database (https://www.ezbiocloud.net/). Phylogenetic analysis was performed by using Molecular Evolutionary Genetics Analysis 11 (MEGA 11)^44^. Sequences of B3-10^T^ and related type species were used to construct a phylogenetic tree by the neighbour-joining (NJ)^45^, maximum-likelihood (ML)^46^, and maximum-parsimony (MP)^47^ algorithms with the Kimura 2-parameter calculation model^48^. Bootstrap values were calculated based on 1000 replications^49^.

Based on comparative analysis of 16S rRNA gene sequences and phylogenetic analysis, reference strains closely related to strain B3-10^T^ were selected. The reference strains *Arachidicoccus soli* KCTC 92782^T^ (= KIS59-12^T^), *Arachidicoccus rhizosphaerae* KCTC 22378^T^ (= Vu-144^T^), *Arachidicoccus terrestris* KCTC 92783^T^ (= 5Gh13-10^T^), *Arachidicoccus ginsenosidivorans* KCTC 22820^T^ (= Gsoil 809^T^), and *Haoranjiania flava* KCTC 42956^T^ (= LIP-5^T^) were obtained from Korean Collection for Type Cultures (KCTC) and used for physiological and chemotaxonomic studies.

### Whole-genome sequencing and phylogenomic analysis

For whole genome sequencing of strain B3-10^T^, genomic DNA was extracted using the MG Genomic DNA purification kit (MGmed). The extracted gDNA was sequenced using the Pacific Biosciences (PacBio) RSII sequencing platform using 20 kb SMRTbell template library and de novo assembly by Macrogen Inc. Hierarchical Genome Assembly Process (HGAP) 3 Analysis Application software (https://www.pacb.com/products-and-services/analytical-software/smrt-analysis/) was used for genome assembly. The gene annotation of each CDSs was performed with homology searches against Prokka v1.12b and eggNOG 4.5 databases^50,51^.

The G + C content was calculated based on the whole-genome sequence. Genomic similarities between strain B3-10^T^ and related reference strains were quantified by average nucleotide identity (ANI), average amino acid identity (AAI), and digital DNA-DNA hybridization (dDDH). ANI values were calculated employing CJ Bioscience’s online orthologous ANI tool^52^; AAI values were calculated by using an online tool developed by Kostas group at Georgia Institute of Technology (http://enve-omics.ce.gatech.edu/aai/)^53^; and dDDH values were calculated using Genome-to-Genome Distance Calculator (GGDC 2.1; http://ggdc.dsmz.de/ggdc.php/)^54^.

Phylogenomic analysis based on whole genome was reconstructed using the Reference sequence Alignment based Phylogeny builder (REALPHY) free online pipeline (https://realphy.unibas.ch/realphy/), which was based on phyML, maximum likelihood methods from the aligned SNP position^55^.

### Phenotypic characteristics and chemotaxonomy

Cell morphology was observed with cells grown in R2A medium at 30 °C for 3 days by scanning electron microscopy and transmission electron microscopy. The temperature range for growth was determined on R2A agar at 4, 10, 15, 20, 25, 30, 37, 45, and 55 °C for 7 days. The pH range for growth was determined in R2A media with pH 4.0, 5.0–9.0 (in increments of 0.5 pH units) at 30 °C for 7 days, using the biological buffer. The following biological buffers were used to adjust the pH: C_6_H_8_O_7_/Na_2_HPO_4_.7H_2_O for pH 4.0–6.0 and NaH_2_PO_4_/Na_2_HPO_4_.7H_2_O for pH 6.5–7.5 and C_8_H_11_N_2_NaO_3_/HCl for pH 8.0–9.0 and NH_2_CH_2_COOH/NaOH for pH 10.0. NaCl tolerance was presenced on R2A with 0–5.0% NaCl (at intervals of 0.5%) at 30 °C for 7 days. Anaerobic growth was tested after incubation on R2A agar with a GasPak anaerobic system (BBL) at 30 °C for 14 days. Additional physiological and biochemical properties were tested using the API 20NE, API ZYM, and API 20E kits (bioMérieux). Catalase activity was determined by the production of bubbles after the addition of a drop of 3.0% (v/v) H_2_O_2_ and oxidase activity was determined using oxidase reagent (bioMérieux).

For most experiments, strain B3-10^ T^ and the reference strains were cultivated on R2A agar at 30 °C for 3 days, unless stated otherwise. Cellular fatty acids were analyzed using protocol by Sasser^56^, and gas chromatography with TSBA database version 6.1 was used. The isoprenoid quinones and polar lipids of strain B3-10^T^ and the reference strains were extracted from freeze-dried cells. The isoprenoid quinone was extracted using chloroform/methanol (2:1; v/v) and purified using TLC on a Kieselgel 60 F254 plate with petroleum benzene / diethyl ether (9:1; v/v) as solvent. Purified extracts were identified using high-performance liquid chromatography (HPLC) analysis as described by Komagata and Suzuki^57^. Polar lipids were analyzed according to Minnikin et al*.*^58^ and identified with 50% H_2_SO_4_, 0.25% ninhydrin, and molybdenum blue spray reagent.

### Plant growth-promoting characteristics

ACC deaminase activity of the strain B3-10^T^ was determined by measuring the amount of α-ketobutyrate, a product of ACC cleavage by ACC deaminase^59,60^. All measurements were performed in triplicate. To induce the ACC deaminase activity of strain B3-10^T^, it was cultured for 48 h at 28 °C in 30 ml DF (Dworkin and Foster) minimal salts media^61^ (DF salts per liter: 4.0 g KH_2_PO_4_, 6.0 g Na_2_HPO_4_, 0.2 g MgSO_4_.7H_2_O, 2.0 g glucose, 2.0 g gluconic acid and 2.0 g citric acid with trace elements: 1 mg FeSO_4_.7H_2_O, 10 mg H_3_BO_3_, 11.19 mg MnSO_4_.H_2_O, 124.6 mg ZnSO_4_.7H_2_O, 78.22 mg CuSO_4_.5H_2_O, 10 mg MoO_3_, pH 7.2) supplemented with 5 mM final concentration of ACC, and the negative control was cultured in DF medium without ACC. The cultured bacterial cells were harvested by centrifugation at 12,000 g for 10 min and washed twice with 0.1 M Tris–HCl (pH 7.5) to remove medium. The cell pellet was then resuspended in 200 μl of 0.1 M Tris–HCl (pH 8.5), after which 10 μl of toluene was added and the suspension was vortexed at high speed for 30 s. Toluenized cell suspension (50 μl) was placed in each 1.5 ml tube, 5 μl of 0.5 M ACC was added only to the positive control sample, and then all samples were incubated at 28 °C for 30 min. As a blank, a tube containing 5 μl of 0.5 M ACC and 50 μl of 0.1 M Tris–HCl (pH 8.5) was incubated under the same conditions. The remaining toluenized cell suspension was used to determine protein concentration according to the method of Bradford^62^. 500 μl of supernatant was transferred to a glass test tube and mixed with 400 μl of 0.56 N HCl. Following that, 150 μl of the 2,4-dinitrophenylhydrazine reagent (0.2% 2,4-dinitrophenylhydrazine in 2 M HCl) was added and vortexed, and the mixture was incubated at 28 °C for 30 min. Finally, 1 ml of 2 N NaOH was added, mixed and the absorbance was measured at 540 nm. Assessment of ACC deaminase activity was compared to the α-ketobutyrate standard curve, and the units of ACC deaminase activity were expressed as nmol α-ketobutyrate mg protein.

Siderophore production was performed on CAS agar using the diffusion assay method^63^. The strain B3-10^ T^ was cultured in DF salts broth^61^ for 5 days, then 100 μl of the supernatant was dropped onto a disc placed on CAS agar and the development of an orange halo around the disc was observed.

To test PGP ability, 48 *Nicotiana benthamiana* seeds were surface-sterilized with 70% (v/v) ethanol for 3 min, followed by a 2.5% (w/v) sodium hypochlorite for 15 min. After washing three times for 5 min in sterile distilled water, each six seeds were germinated on one 0.5X Murashige and Skoog (MS; Duchefa) agar plate each. Four of the eight plates were used as control and the other four plates were inoculated with the strain B3-10^T^ about 5 cm away from the tip of the seeds. The seeds were grown in a growth chamber at 25 ± 1 °C a 16-h light/8-h dark cycle for 14 days. After 14 days, fresh weight, root length, and total chlorophyll content were measured and evaluated. Total chlorophyll content was measured in extracts in 100% ethanol and calculated as described by Batool et al.^64^ All experiments were repeated three times to obtain average values and the data were statistically analyzed.

### Supplementary Information

Supplementary Information 1.

Supplementary Table S4.

## Data Availability

The 16S rRNA gene sequences of strain B3-10^T^ have been deposited in GenBank under the accession number OP693468. The complete genome sequences of a chromosome and a plasmid of strain B3-10^T^ have been deposited in the GenBank database under accession numbers CP044016 (chromosome) and CP044017 (plasmid), respectively.
