# Supplementary material for: A novel plant growth-promoting rhizobacterium, Rhizosphaericola mali gen. nov., sp. nov., isolated from healthy apple tree soil
Source: Sci Rep. 2024 Jan 10;14:1038. doi: 10.1038/s41598-024-51492-y (PMC10781739; doi:10.1038/s41598-024-51492-y)
Supplement: Supplementary file 1 — Supplementary Information 1. [file 41598_2024_51492_MOESM1_ESM.pdf]

Supplementary Information

A novel plant growth-promoting rhizobacterium, *Rhizosphaericola mali* gen. nov., sp. nov., isolated from healthy apple tree soil

Han Sol Kim[1,2§], Ji-Sun Kim[1§], Min Kuk Suh[1,2], Mi Kyung Eom[1], Jiyoung Lee[1], and Jung-Sook Lee[1,3*]

[1]Korean Collection for Type Cultures, Korea Research Institute of Bioscience and Biotechnology, 181 Ipsin-gil, Jeongeup-si, Jeollabuk-do 56212, Republic of Korea

[2]Department of Lifestyle Medicine, Jeonbuk National University, 79 Gobong-ro, Iksan-si, Jeollabuk-do, 54596 Republic of Korea

[3]University of Science and Technology (UST), 217 Gajeong-ro, Yuseong-gu, Daejeon 34113, Republic of Korea

[§] These authors contributed equally to this work

*** Corresponding author**

Dr. Jung-Sook Lee

Korean Collection for Type Cultures,

Korea Research Institute of Bioscience and Biotechnology,

Jeongeup-si, Jeollabuk-do 56212

Republic of Korea

Tel: +82-63-570-5618, Fax: +82-63-570-5609

E-mail: jslee@kribb.re.kr

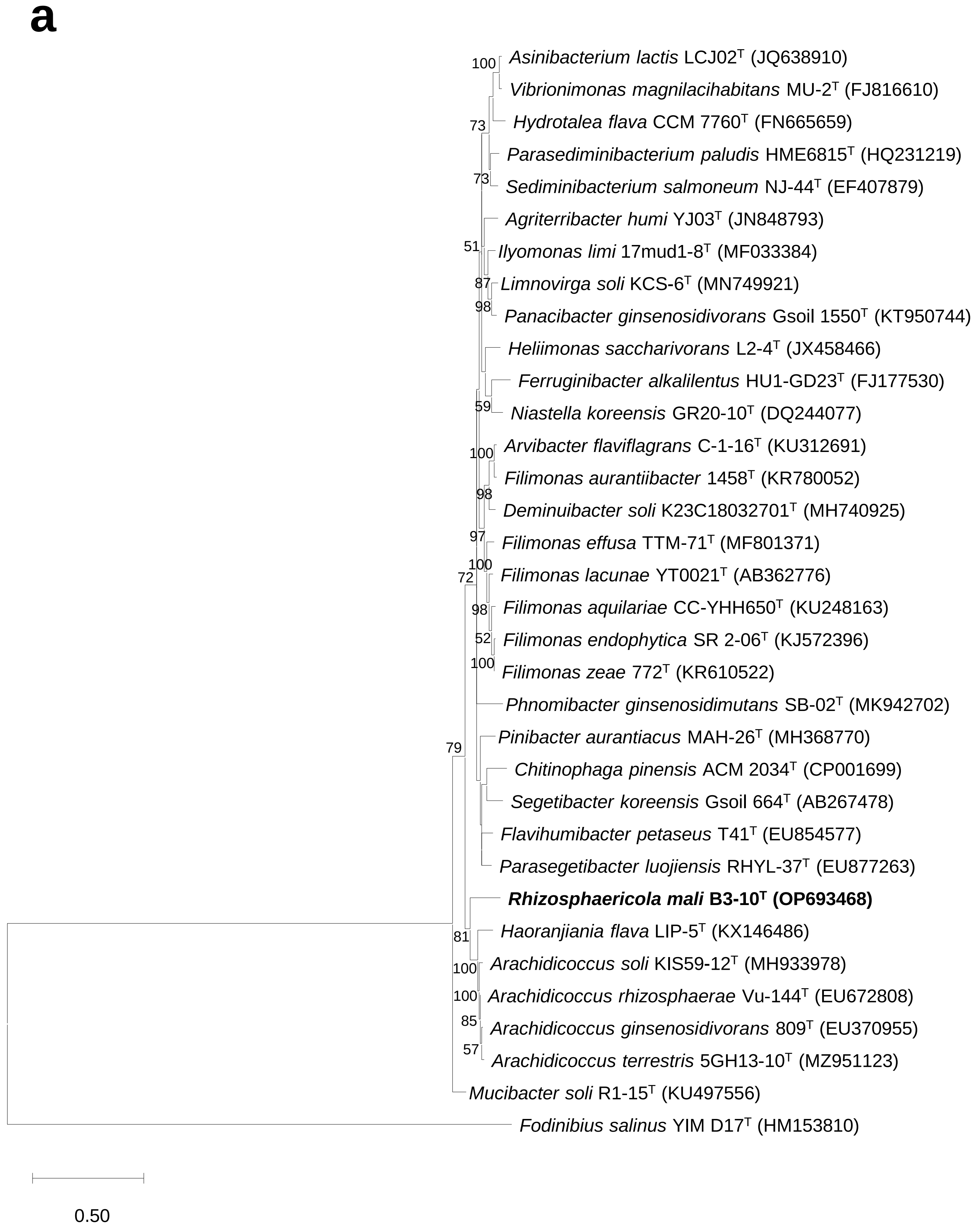
a
Asinibacterium lactis LCJ02T (JQ638910)
Vibrionimonas magnilacihabitans MU-2T (FJ816610)
Hydrotalea flava CCM 7760T (FN665659)
Parasediminibacterium paludis HME6815T (HQ231219)
Sediminibacterium salmoneum NJ-44T (EF407879)
Agriterribacter humi YJ03T (JN848793)
Ilyomonas limi 17mud1-8T (MF033384)
Limnovirga soli KCS-6T (MN749921)
Panacibacter ginsenosidivorans Gsoil 1550T (KT950744)
Heliimonas saccharivorans L2-4T (JX458466)
Ferruginibacter alkalilentus HU1-GD23T (FJ177530)
Niastella koreensis GR20-10T (DQ244077)
Arvibacter flaviflagrans C-1-16T (KU312691)
Filimonas aurantiibacter 1458T (KR780052)
Deminuibacter soli K23C18032701T (MH740925)
Filimonas effusa TTM-71T (MF801371)
Filimonas lacunae YT0021T (AB362776)
Filimonas aquilariae CC-YHH650T (KU248163)
Filimonas endophytica SR 2-06T (KJ572396)
Filimonas zeae 772T (KR610522)
Phnomibacter ginsenosidimutans SB-02T (MK942702)
Pinibacter aurantiacus MAH-26T (MH368770)
Chitinophaga pinensis ACM 2034T (CP001699)
Segetibacter koreensis Gsoil 664T (AB267478)
Flavihumibacter petaseus T41T (EU854577)
Parasegetibacter luojiensis RHYL-37T (EU877263)
Rhizosphaericola mali B3-10T (OP693468)
Haoranjiania flava LIP-5T (KX146486)
Arachidicoccus soli KIS59-12T (MH933978)
Arachidicoccus rhizosphaerae Vu-144T (EU672808)
Arachidicoccus ginsenosidivorans 809T (EU370955)
Arachidicoccus terrestris 5GH13-10T (MZ951123)
Mucibacter soli R1-15T (KU497556)
Fodinibius salinus YIM D17T (HM153810)
100
73
73
51
87
98
59
100
98
97
100
72
98
52
100
79
81
100
100
85
57
0.50

b

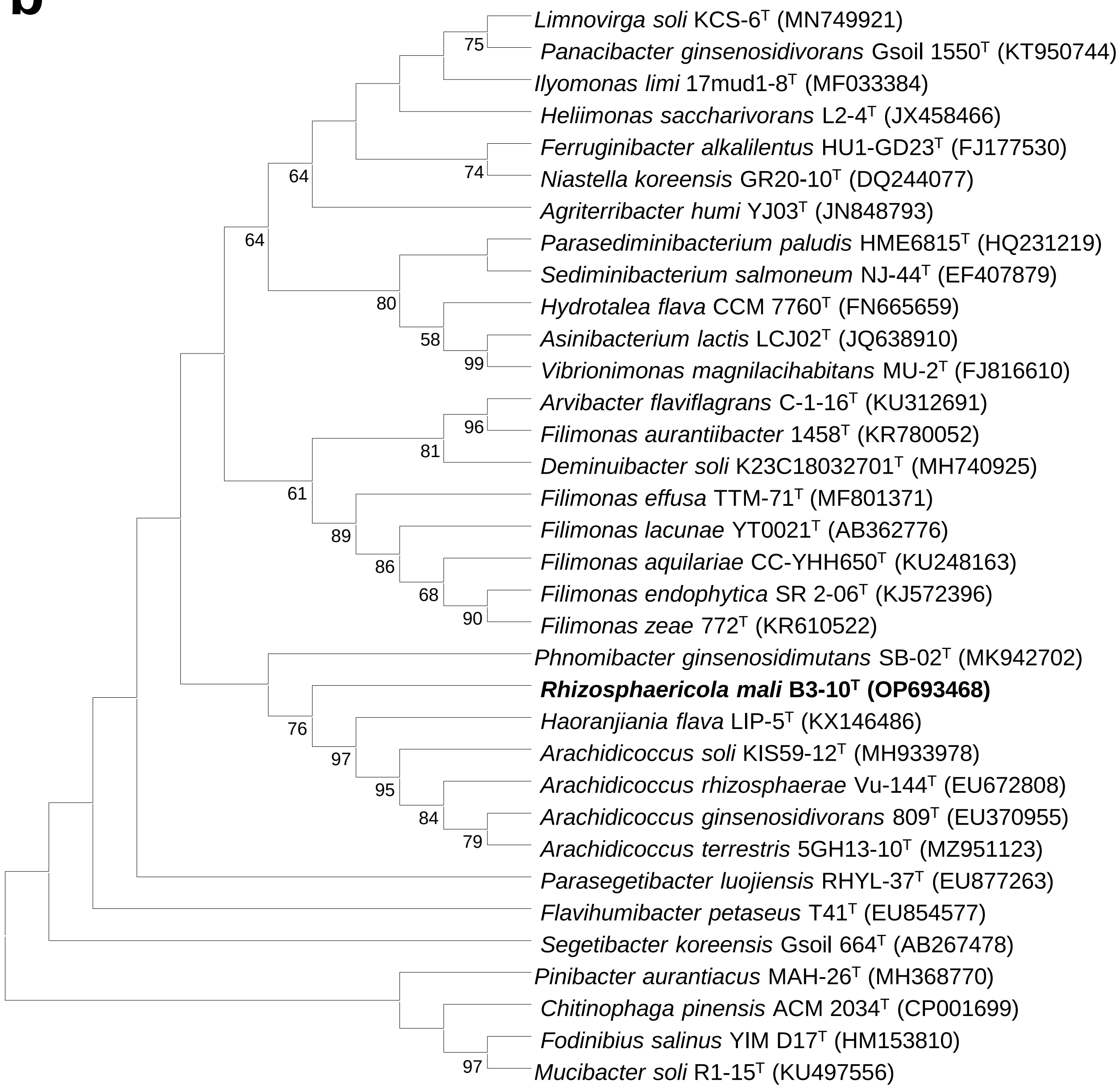

Fig. S1 Maximum-likelihood (a) and maximum-parsimony (b) trees showing the phylogenetic relationships between strain B3-10^{T} and their closely related taxa, based on 16S rRNA gene sequences. Bootstrap values over 50% are shown on the nodes as percentages of 1,000 replicates. *Fodinibius salinus* YIM D17^{T} was used as an outgroup.

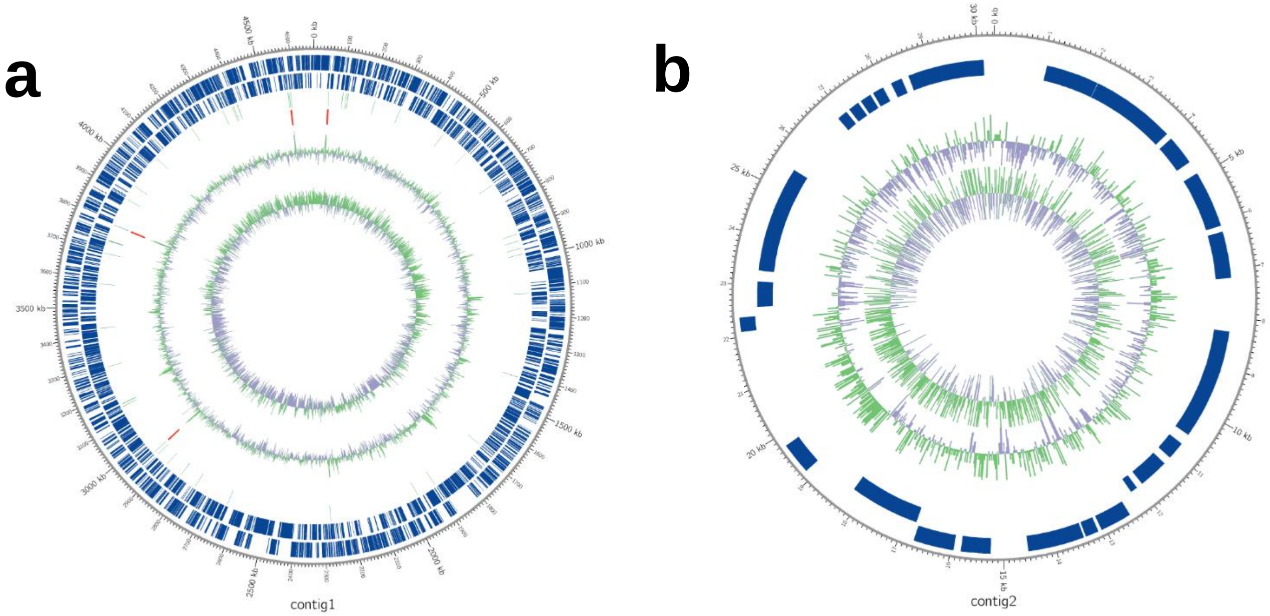

Fig. S2 Graphical circular map of strain B3-10^{T}. Circular map was drawn by applying contig′s annotation result. (a) chromosome; (b) plasmid. Marked characteristics are shown from outside to the center; CDS on forward strand, CDS on reverse strand, tRNA, rRNA, GC content, and GC skew.

Forward CDS, region that is not CDS is described as blank; Reverse CDS, region that is not CDS is described as blank; tRNA, Region of tRNA is marked in light green; rRNA, Region of rRNA is marked in red; GC content, Region that has higher value of GC percentage than that of average is described in exterior light green peak. Region that has lower value of GC percentage than that of average is described in interior lavender peak. Height of the peak describes the difference from the average GC percentage.; GC skew, According to the formula, (G-C)/(G+C), positive value shows that G is dominant while negative value shows that C is dominant. The exterior light green peak describes the region that has higher G content while interior lavender peak describes the region that higher C content.

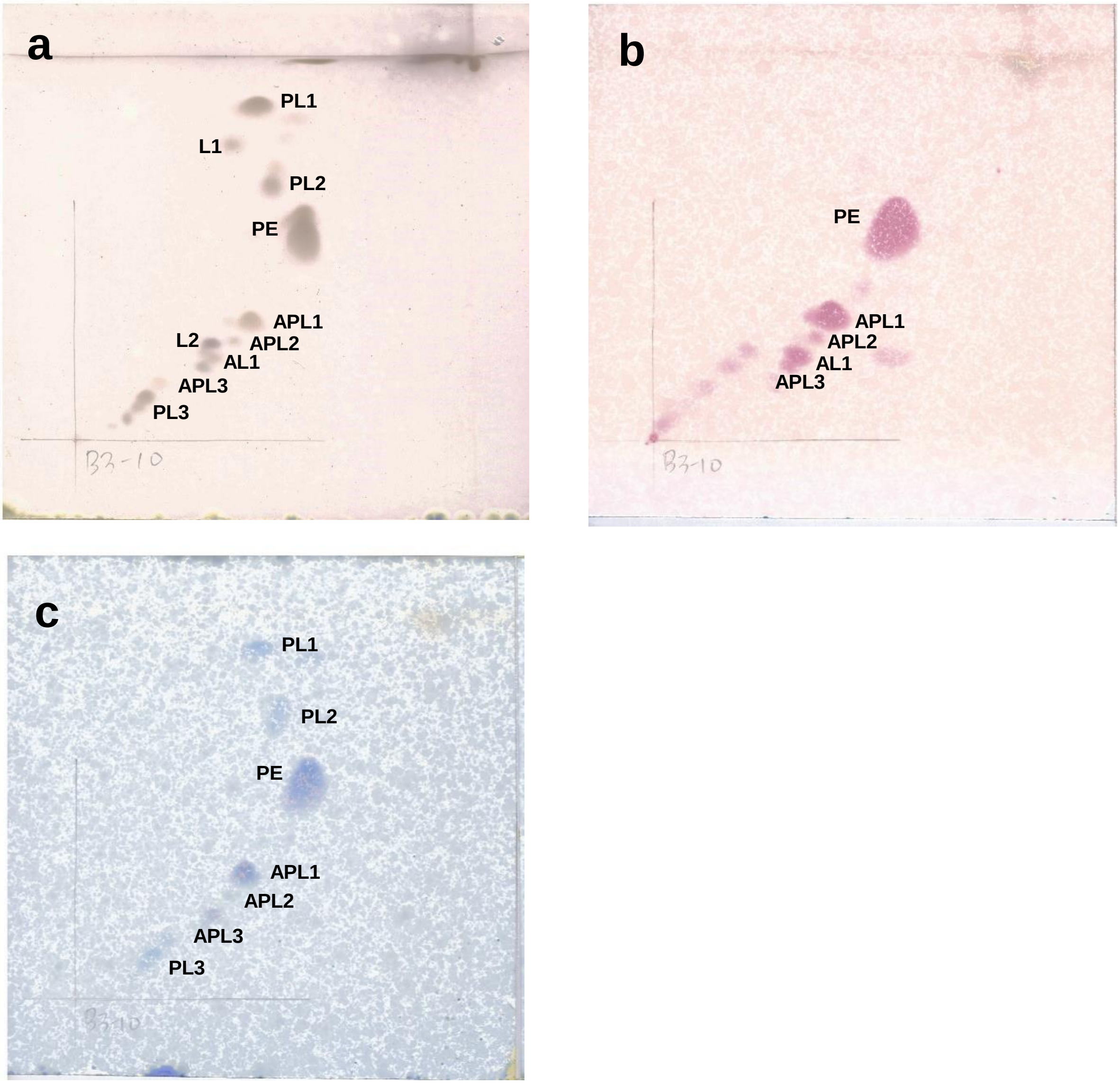

Fig. S3 The polar lipid profiles of strain B3-10^T after staining with 50 % H_2SO_4 (a), ninhydrin (b) and molybdenum blue reagent (c).

PE, phosphomolybdic acid; PL, unidentified phospholipids; APL, unidentified aminophospholipids; AL, unidentified aminolipids; L, unidentified lipids.

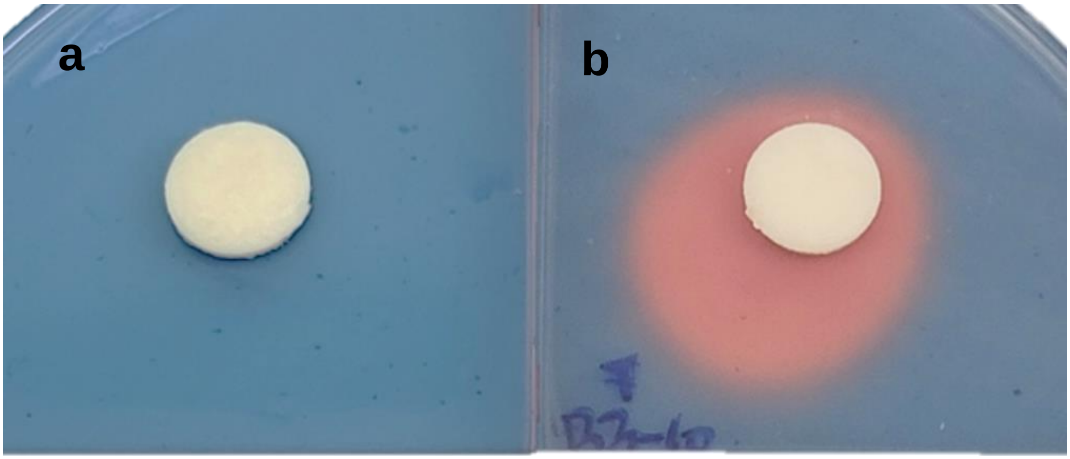

Fig. S4 Siderophore production on CAS agar of strain B3-10^{T}

(a), DF salts broth; (b), supernatant of strain B3-10^{T} cultured for 5 days in DF salt broth.

Supplementary Table S1 Genomic features of strain B3-10^T

Attributes	
**BioSample No.**	SAMN11047026
**Accession No.**	NZ_CP044016 (chromosome) and NZ_CP044017 (plasmid)
**Genome features**	
**Genome size (bp)**	4,701,441
**Number of contigs**	1
**Genome Coverage**	423.0x
**G + C content (mol%)**	34.2
**No. of CDSs**	4,033
**Pseudo genes**	21
**tRNA**	53
**rRNA**	12

Supplementary Table S2 Results of ANI, dDDH, and AAI between genomes of strain B3-10^{T} and other type species in the family *Chitinophagaceae*

**Reference strains**	**NCBI Accession No.**	**ANI (%)**	**dDDH (%)**	**AAI (%)**
Arachidicoccus soli KIS59-12^{T}	CP032489	67.0	22.3	56.6
Hydrotalea flava CCUG 51397^{T}	LUHG01000000	66.6	18.7	53.6
Panacibacter ginsenosidivorans Gsoil1550^{T}	CP042435	66.6	21.8	53.6
Sediminibacterium salmoneum NBRC 103935^{T}	AXZP01000000	66.5	23.6	53.3
Limnovirga soli KCS-6^{T}	WHPF00000000	66.2	19.9	52.9
Lacibacter cauensis CGMCC 1.7271^{T}	VLLE01000000	65.8	21.8	51.2
Gynurincola endophyticus HBUM179779^{T}	RCWL01000000	65.5	22.3	51.4
Parafilimonas terrae DSM 28286^{T}	FOXQ01000000	65.5	26.2	53.4
Arachidicoccus ginsenosidivorans Gsoil 809^{T}	CP042434	65.4	21.6	52.7
Ilyomonas limi 17mud1-8^{T}	SZQL01000000	65.4	24.5	53.6
Segetibacter koreensis DSM 18137^{T}	KB893314	65.4	22.3	52.7
Filimonas lacunae NBRC 104114^{T}	AP017422	65.3	24.8	54.3
Haoranjiania flava LIP-5^{T}	JAOTPL000000000	65.3	25.3	46.0
Arachidicoccus rhizosphaerae Vu-144^{T}	FNQY01000000	65.2	18.6	54.0
Edaphocola aurantiacus H2^{T}	VOSI01000000	65.2	27.9	48.2
Pinibacter aurantiacus MAH-26^{T}	JAHSPG010000000	65.2	25.3	52.5
Arachidicoccus terrestris 5GH13-10^{T}	CP083387	65.2	21.7	54.2
Hydrobacter penzbergensis DSM 25353^{T}	FNNO01000000	65.1	23.2	54.1
Deminuibacter soli K23C18032701^{T}	QTJU01000000	65.0	25.7	53.7
Agriterribacter humi YJ03^{T}	VOHQ01000000	64.9	24.9	50.8
Phnomibacter ginsenosidimutans SB-02^{T}	CP046566	64.9	26.9	49.4
Terrimonas ferruginea DSM 30193^{T}	KE384398	64.6	29.7	51.2

Niabella aurantiaca DSM 17617^T	KB893625	64.5	29.2	50.4
Flavipsychrobacter stenotrophus RB1R16^T	PPSL01000000	64.2	29.2	46.8
Niastella koreensis GR20-10^T	CP003178	64.2	26.6	51.1
Paraflavitalea soli 5GH32-13^T	CP032157	64.2	25.4	39.0
Chitinophaga pinensis DSM 2588^T	CP001699	64.1	27.9	50.2
Paracnuella aquatica N24^T	QOWJ01000000	64.1	32.1	50.7
Pseudobacter ginsenosidimutans DSM 18116^T	SGXA01000000	64.0	28.6	51.1
Pseudoflavitalea rhizosphaerae KACC 18655^T	RCSU00000000	64.0	30.9	51.0
Flavihumibacter petaseus NBRC 106054^T	BBWV01000000	63.8	27.1	51.1
Cnuella takakiae DSM 26897^T	FQUO01000000	63.7	32	50.4
Puia dinghuensis CGMCC 1.15448^T	BMJC01000000	63.1	28.3	50.9
Thermoflavifilum aggregans DSM 27268^T	PGFG01000000	63.1	23.5	49.1
Thermoflavifilum thermophilum DSM 14807^T	FPCJ01000000	63.1	25.2	49.1
Dinghuibacter silviterrae DSM 100059^T	SODV01000000	62.4	28.5	50.5

Supplementary Table S3 Differential characteristics of strain B3-10^T and the type strains of closely related species

**Characteristics**	**1**	**2**	**3**	**4**	**5**	**6**
**Optimal pH**	7.0	7.0	6.5	7.0	7.0	7.0
**Optimal temperature (°C)**	30	30	30	30	30	25
**NaCl concentration (%)**	0	0	0	0	0	0
**API 20NE**						
L-arabinose	+	+	-	+	-	-
D-mannose	+	+	+	+	-	-
D-mannitol	-	-	-	+	-	-
N-acetyl-D-glucosamine	+	+	+	+	+	-
D-maltose	+	+	+	+	-	+
**API ZYM**						
esterase (C4)	+	+	-	-	-	-
valine arylamidase	+	+	w	w	-	+
trypsin	w	-	-	-	-	+
β-galactosidase	+	w	+	-	+	-
α-glucosidase	+	-	-	-	-	+
β-glucosidase	+	+	-	-	-	-
α-mannosidase	-	-	-	-	-	+
α-fucosidase	+	+	+	-	-	+
**API 20E**						
gelatin	w	-	+	w	-	-
D-glucose	+	+	-	+	w	+
D-melibiose	+	+	+	+	-	+
amygdalin	+	w	+	-	+	+
L-arabinose	+	+	-	+	-	-

Strains: 1, *Rhizosphaericola mali* B3-10^T; 2, *Arachidicoccus soli* KCTC 92782^T 3, *Arachidicoccus rhizosphaerae* KCTC 22378^T; 4, *Arachidicoccus terrestris* KCTC 92783^T, 5, *Arachidicoccus ginsenosidivorans* KCTC 22820^T; 6, *Haoranjiania flava* KCTC 42956^T. All data were obtained in this study. +, Positive; w, weakly positive; -, negative.

Supplementary Table S4 Morphological and physiological characteristics between strain B3-10^{T} and other type species in the family *Chitinophagaceae*

The table can be viewed in the separately attached Excel file.

Strains: 1. B3-10^{T}; 2, *Agriterribacter humi* YJ03^{T}; 3, *Arachidicoccus rhizosphaerae* Vu-144^{T}, 4, Aridibaculum aurantiacum SYSU D00508^{T}; 5, *Arvibacter flaviflagrans* C-1-16^{T}; 6, Aurantisolimonas haloimpatiens BN130233T; 7, Chitinophaga pinensis UQM 2034^{T}; 8, Cnuella takakiae RG1-1^{T}; 9, Compostibacter hankyongensis BS27^{T}; 10, Deminuibacter soli K23C18032701^{T}; 11, Dinghuibacter silviterrae DHOA34^{T}; 12, Edaphobaculum flavum 1-116^{T}; 13, Edaphocola aurantiacus H2^{T}; 14, Ferruginibacter alkalilentus HU1-HG42^{T}; 15, Filimonas lacunae YT0021^{T}; 16, Flaviaestuariibacter amylovorans GCR0105^{T}; 17, Flavihumibacter petaseus T41^{T}; 18, Flavipsychrobacter stenotrophus RB1R16^{T}; 19, Flavisolibacter ginsengiterrae Gsoil 492^{T}; 20, Flavitalea populi HY-50R^{T}; 21, Gynurincola endophyticus HBUM179779^{T}; 22, Haoranjiania flava LIP-5^{T}; 23, Heliimonas saccharivorans L2-4^{T}; 24, Hydrobacter penzbergensis EM 4^{T}; 25, Hydrotalea flava CCUG 51397^{T}; 26, Ilyomonas limi 17mud1-8^{T}; 27, Lacibacter cauensis NJ-8^{T}; 28, Limnovirga soli KSC-6^{T}; 29, Mucibacter soli R1-15^{T}; 30, Nemorincola caseinilytica J116-2^{T}; 31, Niabella aurantiaca R2A15-11^{T}; 32, Niastella koreensis GR20-10^{T}; 33, Niveitalea solisilvae 6-4^{T}; 34, Panacibacter ginsenosidivorans Gsoil 1550^{T}; 35, Paracnuella aquatic N24^{T}; 36, Parafilimonas terrae 5GHs7-2^{T}; 37, Paraflavisolibacter caeni LB-8^{T}; 38, Paraflavitalea soli 5GH32-13^{T}; 39, Parapseudoflavitalea muciniphila PMP191F^{T}; 40, Parasediminibacterium paludis HME6815^{T}; 41, Parasegetibacter luojiensis RHYL-37^{T}; 42, Phnomibacter ginsenosidimutans SB-02^{T}; 43, Pinibacter aurantiacus MAH-26^{T}; 44, Pseudobacter ginsenosidimutans ; 45, Pseudoflavitalea rhizosphaerae T16R-265^{T}; 46, Puia dinghuensis 4GSH07^{T}; 47, Rurimicrobium arvi J107-1^{T}; 48, Sediminibacterium salmoneum NJ-44^{T}; 49, Segetibacter koreensis Gsoil 664^{T}; 50, Taibaiella smilacinae PTJT-5^{T}; 51, Terrimonas ferruginea IAM 15098^{T}; 52, Thermoflavifilum aggregans P373^{T}.

2, (Lee and Whang 2020); 3, (Madhaiyan et al. 2015); 4, (Dong et al. 2023); 5, (Chaudhary and Kim 2016); 6, (Liu et al. 2018a); 7, (Sangkhobol and Skerman 1981); 8, (Zhao et al. 2014); 9, (Siddiqi et al. 2016a); 10, (Wang et al. 2019a); 11, (Lv et al. 2016); 12, (Cao et al. 2017); 13, (Choi et al. 2019); 14, (Lim et al. 2009); 15, (Shiratori et al. 2009); 16, (Kang et al. 2015); 17, (Zhang et al. 2010); 18, (Liu et al. 2018b); 19, (Yoon and Im 2007); 20, (Wang et al. 2011); 21, (Zhang et al. 2019); 22, (Zhang et al. 2016); 23, (Leandro et al. 2013); 24, (Eder et al. 2015); 25, (Kämpfer et al. 2011); 26, (Chhetri et al. 2019); 27, (Qu et al. 2009); 28, (Yim et al. 2021); 29, (Kim et al. 2019); 30, (Chaudhary et al. 2018b; Chaudhary et al. 2018a); 31, (Kim et al. 2007); 32, (Weon et al. 2006); 33, (Hyeon et al. 2017); 34, (Siddiqi et al. 2016b); 35, (Wang et al. 2019b); 36, (Kim et al. 2014); 37, (Yuan et al. 2023); 38, (Heo et al. 2020); 39, (Lawson et al. 2020); 40, (Kang et al. 2016); 41, (Zhang et al. 2009); 42, (Siddiqi et al. 2021); 43, (Huq et al. 2021); 44, (Siddiqi and Im 2016); 45, (Kim et al. 2016); 46, (Lv et al. 2017); 47, (Dahal et al. 2017); 48, (Qu and Yuan 2008); 49, (An et al. 2007); 50, (Zhang et al. 2013); 51, (Xie and Yokota 2006); 52, (Anders et al. 2014)

DPG, diphosphatidylglycerol; PE, phosphatidylethanolamine; PG, phosphatidylglycerol; PIM, phosphatidyl-inositolmannosides; AGL, aminoglycolipid; PG, phosphatidylglycerol, GPL, glycophospholipid ; AGL, aminoglycolipid ; APL, aminophospholipid; AL, unidentified aminolipids; PL, unidentified phospholipid; L, unidentified lipids; -, not detected.

Supplementary Table S5 Cellular fatty acid profiles (% of total) of strain B3-10[T] and the type strains of closely related members of the family *Chitinophagaceae*

**Fatty acid***	**1**	**2**	**3**	**4**	**5**	**6**
**Saturated**						
$C_{13:1}$ (at 12-13)	TR	-	TR	-	TR	-
$C_{14:0}$	TR	TR	TR	TR	1.1	-
$C_{16:0}$	2.9	2.7	-	3.7	**10.0**	1.7
$C_{17:1}$ ω6c	-	-	-	TR	-	1.2
**Branched**						
iso-$C_{14:0}$	-	-	-	-	-	2.7
iso-$C_{15:0}$	**25.7**	**38.9**	**45.9**	**26.6**	**42.4**	**33.5**
$C_{15:0}$ 2OH	-	-	-	-	-	1.0
iso-$C_{15:0}$ 3OH	-	5.6	1.9	2.0	-	2.8
iso-$C_{15:1}$ G	**25.5**	**18.2**	**10.3**	**21.8**	**14.5**	1.4
iso-$C_{15:1}$ 3OH	-	-	-	-	2.5	-
anteiso-$C_{15:0}$	1.6	1.8	1.2	2.5	1.1	**27.3**
anteiso-$C_{15:1}$ A	TR	-	-	-	-	3.0
iso-$C_{16:0}$	-	-	7.2	TR	-	4.2
iso-$C_{16:0}$ 3OH	-	TR	TR	-	-	4.2
iso-$C_{17:0}$	-	-	TR	-	-	-
iso-$C_{17:0}$ 3OH	4.8	**18.4**	**16.5**	**16.8**	**14.2**	-
**Hydroxy**						
$C_{16:0}$ 2OH	-	-	TR	2.3	TR	-
$C_{16:0}$ 3OH	9.7	3.1	2.8	3.9	3.6	TR
$C_{17:0}$ 2OH	-	1.0	TR	TR	-	2.2
$C_{17:0}$ 3OH	-	-	-	-	-	6.5
**Summed Feature3**	**28.0**	9.2	**11.0**	**18.1**	9.1	7.7

Strains: 1, *Rhizosphaericola mali* B3-10[T]; 2, *Arachidicoccus soli* KCTC 92782[T] 3, *Arachidicoccus rhizosphaerae* KCTC 22378[T]; 4, *Arachidicoccus terrestris* KCTC 92783[T], 5, *Arachidicoccus ginsenosidivorans* KCTC 22820[T]; 6, *Haoranjiania flava* KCTC 42956[T]. All data were obtained in this study. TR, trace (<1%); -, not detected.

* Values are percentages of total fatty acids. Data are from this study. Fatty acid are listed using standard abbreciations (number of carbon atoms: number of double bonds). Summed features represent two or three fatty acids that cannot be separated by GLC using the MIDI system. Summed Feature3 comprised $C_{16:1}\omega6c$ and/or $C_{16:1}\omega7c$

Supplementary Table S6 Various PGP-related genes found in the genome of strain B3-10^T

Plant growth-promoting traits	
dcyD	1-aminocyclopropane-1-carboxylate(ACC) deaminase
rimM	Involved in ACC-deaminase coding. An accessory protein needed during the final step in the assembly of 30S ribosomal subunit, possibly for assembly of the head region.
ipdC	Indole-3-pyruvate decarboxylase/phenylpyruvate decarboxylase of the indole-3-pyruvate pathway for the synthesis of indole acetic acid (IAA)
ysnE	gCN5-related N-acetyltransferase. IAA biosynthetic pathway associated with tryptophan acetyltransferase gene
miaAB	Involved in cytokinin biosynthesis. Encodes tRNA dimethyl allyltransferase, which removes the zeatin precursor from tRNA
ipdC	involved in phenylacetic acid (PAA) biosynthesis. Pyruvate decarboxylase
speA	involved in Polyamines biosynthesis. Agmatine synthesis
speE	involved in Polyamines biosynthesis. O-methyltransferase. Catalyzes the production of spermidine from putrescine and decarboxylated S-adenosylmethionine (dcSAM), which acts as an aminopropyl donor.
speH	involved in Polyamines biosynthesis. Catalyzes the decarboxylation of S-adenosylmethionine to S-adenosylmethioninamine (dcAdoMet). Propylamine donor required for synthesis of polyamines spermine and spermidine from diamine putrescine
metK	involved in Polyamines biosynthesis. Catalyzes the formation of S-adenosylmethionine from methionine and ATP
kdpA	The components of the high-affinity ATP-driven potassium transport (or KDP) system, which catalyzes the hydrolysis of ATP coupled with the exchange of hydrogen and potassium ions
kdpB	The components of the high-affinity ATP-driven potassium transport (or KDP) system, which catalyzes the hydrolysis of ATP coupled with the exchange of hydrogen and potassium ions
kdpC	The components of the high-affinity ATP-driven potassium transport (or KDP) system, which catalyzes the hydrolysis of ATP coupled with the exchange of hydrogen and potassium ions
kdpD	The components of the high-affinity ATP-driven potassium transport (or KDP) system, which catalyzes the hydrolysis of ATP coupled with the exchange of hydrogen and potassium ions. Histidine kinase

Resistance to drugs and heavy metals	
fetA	Involved in tetracycline resistance. Omologs of the multifunctional tetracycline-metal/H+ antiporter (tetA)
msbA	Antibiotic efflux ABC transporter (ATP-binding protein)
arsC	Involved in arsenic detoxification
fhuA	Ferrichrome-iron receptor
fecI	putative RNA polymerase sigma factor FecI
feoB	Ferrous iron transport protein b
fbpC	Part of the ABC transporter complex ModABC involved in molybdenum import. Responsible for energy coupling to the transport system
exbB	MotA TolQ exbB proton channel
copA	Involved in copper resistance. Multicopper oxidase
arsH	Involved in Arsenic resistance. Nadph-dependent fmn reductase. arsenical resistance protein ArsH
arsC	Involved in Arsenic resistance. Arsenate reductase
arsR	Involved in Arsenic resistance. arsR family transcriptional regulator
cusR	Involved in copper/silver resistance. RegulatoR, two-component system, OmpR family, copper resistance
cusS	Involved in copper/silver resistance. Histidine kinase, two-component system, OmpR family, heavy metal sensor
czcA	Involved in cobalt/zinc/cadmium resistance. Heavy metal efflux pump and cobalt-zinc-cadmium resistance protein CzcA
czcB	Involved in cobalt/zinc/cadmium resistance. Efflux transporter rnd family, mfp subunit. Cobalt-zinc-cadmium resistance protein CzcB
ompW	Involved in cobalt/zinc/cadmium resistance. Outer membrane protein

Plant protection from stress	
PaaE	Plant protection from nitrosative stress. Is involved in NO detoxification in an aerobic process, termed nitric oxide dioxygenase (NOD) reaction that utilizes O(2) and NAD(P)H to convert NO to nitrate
sodA	Plant protection from oxidative stress. Superoxide dismutases. Destroys radicals which are normally produced within the cells and which are toxic to biological systems
katEG	Plant protection from oxidative stress. Bifunctional enzyme with both catalase and broad-spectrum peroxidase activity.
ahpC	Plant protection from oxidative stress. Alkyl hydroperoxide reductase
ahpF	Plant protection from oxidative stress. Thioredoxin reductase
ahpD	Plant protection from oxidative stress. Antioxidant protein with alkyl hydroperoxidase activity. Required for the reduction of the AhpC active site cysteine residues and for the regeneration of the AhpC enzyme activity
tpx	Plant protection from oxidative stress. Could remove peroxides or H(2)O(2)
ggt	Plant protection from oxidative stress. gamma-glutamyl transpeptidas
ohrAR	Plant protection from oxidative stress. Resistance to organic peroxides
soxS	Plant protection from oxidative stress.
oxyR	Plant protection from oxidative stress.

Supplementary Table S7 ACC deaminase activity from strain B3-10^T

	**nmol α-ketobutyrate/protein mg**
**with ACC supplementation**	869.03 ±238.13
**without ACC supplementation**	655.90 ±214.77

Values are mean of three replications. ± shows standard debiation.
